# Inflammatory and immune markers in HIV‐infected older adults on long‐term antiretroviral therapy: Persistent elevation of sCD14 and of proinflammatory effector memory T cells

**DOI:** 10.1111/acel.13681

**Published:** 2022-08-16

**Authors:** Makiko Watanabe, Mladen Jergovic, Lisa Davidson, Bonnie J. LaFleur, Yvonne Castaneda, Carmine Martinez, Megan J. Smithey, Raymond P. Stowe, Elias K. Haddad, Janko Nikolich‐Žugich

**Affiliations:** ^1^ Department of Immunobiology University of Arizona College of Medicine‐Tucson Tucson Arizona USA; ^2^ Arizona Center on Aging University of Arizona College of Medicine‐Tucson Tucson Arizona USA; ^3^ BIO5 Institute University of Arizona Tucson Arizona USA; ^4^ R. Ken Coit College of Pharmacy Univeristy of Arizona Tucson Arizona USA; ^5^ Microgen Laboratories La Marque Texas USA; ^6^ Division of Infectious Diseases and HIV Medicine, Department of Medicine Drexel University Philadelphia Pennsylvania USA

**Keywords:** antiretroviral therapy, HIV, immune aging, sCD14

## Abstract

HIV‐positive patients whose viral loads are successfully controlled by active antiretroviral therapy (ART) show no clinical signs of AIDS. However, their lifespan is shorter compared with individuals with no HIV infection and they prematurely exhibit a multitude of chronic diseases typically associated with advanced age. It was hypothesized that immune system aging may correlate with, and provide useful biomarkers for, this premature loss of healthspan in HIV‐positive subjects. Here, we tested whether the immune correlates of aging, including cell numbers and phenotypes, inflammatory status, and control of human cytomegalovirus (hCMV) in HIV‐positive subjects on long‐term successful ART (HIV+) may reveal increased “immunological age” compared with HIV‐negative, age‐matched cohort (HIV−) in participants between 50 and 69 years of age. Specifically, we expected that younger HIV+ subjects may immunologically resemble older individuals without HIV. We found no evidence to support this hypothesis. While T cells from HIV+ participants displayed differential expression in several differentiation and/or inhibitory/exhaustion markers in different T cell subpopulations, aging by a decade did not pronounce these changes. Similarly, while the HIV+ participants exhibited higher T cell responses and elevated inflammatory marker levels in plasma, indicative of chronic inflammation, this trait was not age‐sensitive. We did find differences in immune control of hCMV, and, more importantly, a sustained elevation of sCD14 and of proinflammatory CD4 and CD8 T cell responses across age groups, pointing towards uncontrolled inflammation as a factor in reduced healthspan in successfully treated older HIV+ patients.

## Graphical abstract

Proinflammatory markers, such as sCD14, in plasma and proinflammatory responses of T cells were elevated in successfully treated HIV+ compared with HIV− older adults age‐independently. The higher number of naïve CD8 T cells, higher expression of inhibitory/exhaustion markers (PD‐1, TIM3, CD57) were detected on T cells of HIV+ with or without stimulation. The higher expression of exhaustion markers decreased with age and differences diminished in older populations.

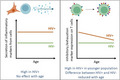

## 1 INTRODUCTION

The development and widespread use of antiretroviral therapy (ART) has reduced AIDS‐associated deaths dramatically since the late 1990s (Palella Jr. et al., 1998). Even though the life expectancy of HIV‐positive individuals increased, they exhibit higher rates of non‐AIDS‐related morbidity and mortality compared with HIV‐negative counterparts (Althoff et al., 2015; Guaraldi et al., 2011; Rasmussen et al., 2015). Active HIV infection causes many abnormal immune phenotypes reminiscent of natural aging, including low CD4/CD8 ratio, chronic inflammation, immune activation, and increase in immunosenescence markers (Kamat et al., 2012; Ronsholt et al., 2013; Sereti et al., 2017). While this analogy is tempting, it is also critically important to remember that HIV initially causes a catastrophic disruption of mucosal immunity and CD4 deficiency (Appay et al., 2007; Brenchley et al., 2006; Yamashita et al., 2001), and while ART helps revert both of these manifestations, it is unclear whether full reconstitution of both parameters is ever achieved. Therefore, most of the HIV‐related phenotypes correlate with virus load or decreased CD4 counts and poor disease prognosis in active HIV infection. Once the ART is initiated, as virus load decrease and CD4 count rises, abnormal immune phenotypes also get back to normal range (Gandhi et al., 2017). However, some of those immunological changes still exist in successfully treated HIV‐positive individuals including impaired memory T cell (van Grevenynghe et al., 2008) and B cell (van Grevenynghe et al., 2011) function, and loss of follicular helper T cell function (Cubas et al., 2015). Those phenotypes in HIV positives appear to be chronic inflammatory responses and they correlate with the functional status of the subjects, which includes parameters such as frailty and the immune risk factors (Castilho et al., 2016).

Immune phenotypes are sensitive to variations based on parameters that include, but are not limited to, age, gender, cytomegalovirus (CMV) serostatus. Human CMV (hCMV) is a widely distributed human herpesvirus that infects 60%–90% of the human population depending on the age and socioeconomic conditions (Cannon et al., 2010; Korndewal et al., 2015; Zuhair et al., 2019). It is a latent persistent infection that is compatible with health in immunocompetent humans, but it represents a substantial threat to immunocompromised, as well as to the developing fetus (Emery, 2001; Istas et al., 1995). It is known that many stressors, including third‐party infection, will reactivate hCMV in a subclinical manner, causing initial viral gene transcription and translation, and, depending on the reactivating stimulus, also DNA replication and viremia (Cook et al., 2002; Forte et al., 2020; Polic et al., 1998; Prosch et al., 2000; Soderberg‐Naucler et al., 2001). Human immune system devotes substantial resources to hCMV control, that can include up to 50% or more of circulating CD8 T effector memory (Tem) cells and T effector memory cells re‐expressing CD45RA (TEMRA) (Munks et al., 2006; Sylwester et al., 2005). No other human microbial pathogen is known to modulate the immune system and host responses more extensively than hCMV—in a monozygotic twin study, it has been shown that 58% of the >100 immune parameters measured were modulated by hCMV infection (Brodin et al., 2015). Therefore, hCMV represents a bellwether measure of immune system fitness and is ideal to assess whether and how this virus is controlled under different physiological situations (Brodin et al., 2015; Rolle & Brodin, 2016; Yan et al., 2021).

The main aim of our study was to investigate whether chronic HIV infection, despite ART therapy, adds years to immune aging and makes immune phenotypes in HIV‐positive individuals. To test this, we recruited HIV‐positive subjects 50–69 years of age, who tested HIV‐positive at least 5 years ago, and who were currently successfully treated with ART (HIV+) as well as HIV‐negative (HIV−) age controls. The impact of controlled HIV infection and age on immune homeostasis and function, including immune responses to hCMV latent infection were assessed in this cohort. To avoid sex at birth bias, we focused on male participants, and took advantage of the fact that nearly all HIV+ participants were CMV seropositive. We compared their immune and inflammatory phenotypes to those of HIV− control groups with similar age, gender and CMV positivity. Consistent with prior studies, we found that the some of the measured parameters were significantly different in HIV+ subjects compared to HIV− controls. While many changes were not age‐sensitive, we found age‐dependent differences as well. Moreover, certain HIV‐associated disturbances persisted in ART‐treated participants into the advanced age, suggesting a prolonged impact of long‐term chronic viral infection on proinflammatory immune status in older adults. These phenotypes may provide clues to overcome morbidity and mortality in successfully treated HIV‐positive populations.

## 2 RESULT

### 2.1 Demographic characteristics of our cohort and study hypothesis

Our cohort consisted of 88 male participants aged 50 and older with 42 HIV+ and 46 HIV− participants, with very close medians between the comparing groups. We selected age of 50 years and older, since the clinical symptoms of immune aging phenotypes become more noticeable after age of 50 and accelerate thenceforth (Katzir et al., 2021). We were not able to recruit enough number of HIV+ participants with age over 70. It is not surprising considering that HIV epidemic started in 1980s in younger populations and the participants with HIV still have about 10 years shorter life expectancy compared with HIV‐counterpart even the recent years (Marcus et al., 2020). All participants were verified to be serologically hCMV+. Demographic features of the cohort are shown in Table 1. HIV+ include less Hispanic or Latino; however, the difference is not statistically significant. Education and annual income are significantly different between HIV+ and HIV− groups. HIV+ and HIV− group has similar comorbidities expect heart attack and liver diseases, which are significantly higher in HIV+ group. All following significant differences in immunological parameters between HIV+ and HIV− were not affected by adjusting for socioeconomic status.

**TABLE 1:** Demographics of study cohort

	HIV+ (n = 42)	HIV− (n = 46)	*p*‐Value
Age, years (Mean ± SD)	59.0 ± 5.3	59.2 ± 5.3	0.850^a^
Years from HIV diagnosis (Mean ± SD)	20.6 ± 7.8	N/A	N/A
Ethnicity, n (%)			
Hispanic or Latino	12 (28.6%)	21 (45.7%)	0.098^b^
NOT Hispanic or Latino	30 (71.4%)	25 (54.3%)	
Race, n (%)			
Native American/Alaska Native	0 (0.0%)	2 (4.4%)	0.890^b^
Asian	0 (0.0%)	3 (6.7%)	
Black or African American	6 (14.6%)	2 (4.4%)	
White	32 (78.0%)	35 (77.8%)	
Other/Not reported	3 (7.1%)	3 (6.5%)	
Education, n (%)			
Middle school	0 (0.0%)	1 (2.2%)	<0.001^b^
High school	12 (28.6%)	6 (13.0%)	
Some college	12 (28.6%)	16 (34.8%)	
Associate degree	6 (14.3%)	3 (6.5%)	
Bachelor's degree	5 (11.9%)	3 (6.5%)	
Master's degree	7 (16.7%)	1 (2.2%)	
Doctoral degree	0 (0.0%)	4 (8.7%)	
Not reported	0 (0.0%)	12 (26.1%)	
Annual income, n (%)			
<$25,000	29 (69.0%)	19 (41.3%)	0.021^b^
$25,000–50,000	5 (11.9%)	8 (17.4%)	
$50,000–75,000	3 (7.1%)	3 (6.5%)	
$75,000–100,000	2 (4.9%)	2 (4.4%)	
>$100,000	2 (4.9%)	2 (4.4%)	
Not reported	1 (2.4%)	13 (28.3%)	
Medical condition/History			
BMI (mean ± SD)	26.7 ± 4.7	27.3 ± 4.7	0.582^a^
Cancer, n (%)	3 (7.1%)	3 (6.5%)	0.908^b^
Diabetes, n (%)	8 (19.0%)	9 (19.6%)	0.626^b^
Heart attack, n (%)	4 (9.5%)	0 (0.0%)	0.032^b^
Stroke, n (%)	2 (4.8%)	0 (0.0%)	0.134^b^
Hypertension, n (%)	18 (42.9%)	18 (39.1%)	0.933^b^
Lung disease, n (%)	7 (16.7%)	3 (6.5%)	0.134^b^
Kidney disease, n (%)	2 (4.8%)	1 (2.2%)	0.504^b^
Liver disease, n (%)	9 (21.4%)	2 (4.4%)	0.016^b^
Arthritis/Joint pain, n (%)	15 (35.7%)	19 (41.3%)	0.591^b^

^a^ Unpaired t‐test.

^b^ Chi‐Square test.

### 2.2 Systematic inflammatory markers in plasma

**FIGURE 1:**
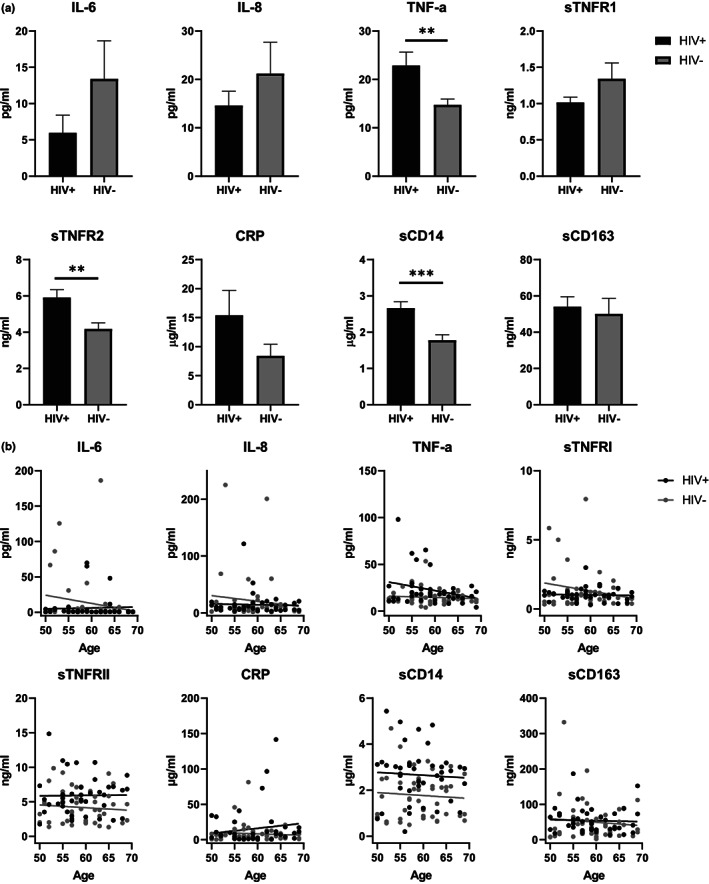
Inflammatory markers in plasma from successfully treated HIV‐positive participants (HIV+) and HIV‐negative counterparts (HIV−). (a) Comparison of inflammatory marker levels in plasma between HIV+ and HIV−. Means and standard errors in each age/HIV group are shown. Significant differences between HIV+ (black) and HC (gray) were determined by unpaired t‐test. *p < 0.05, **p < 0.01, ***p < 0.001. (b) Analysis of covariance analysis of inflammatory markers with age for HIV+ (black dots and lines) and HIV− (gray dots and lines) using an F‐test for the difference in slopes.

Persistent inflammation is one of the common phenotypes for HIV infection and aging and inflammatory markers in plasma are considered to be a good indication of the overall inflammatory status (Bektas et al., 2018; Ferrucci et al., 2002). We measured inflammatory markers in plasma from our cohort, including those reported to show consistent age‐related increase in healthy HIV‐uninfected aging cohorts, such as IL‐6, CRP, soluble (s)TNF receptors 1 and 2, and TNF‐α; as well as those reported to be increased in HIV as potential markers of gut barrier damage and permeability, such as soluble CD163 and soluble CD14. IL‐6, IL‐8, sTNFR1, CRP (trend but no significance), and sCD163 levels were not significantly different between HIV+ and HIV− in both age groups. The mean TNF‐α, sTNFR2 and sCD14 levels were significantly elevated in HIV+ compared with in HIV− (Figure 1a, *p* = 0.0041, *p* = 0.0007, and *p* = 0.0002, respectively). However, the mean levels of those markers by age were not statistically significantly different (Figure 1b). There were no other statistically significant differences in inflammatory markers with increasing age, adjusting for or averaging over HIV status. Of interest, in this cohort, none of the inflammatory markers were significantly higher in the HIV− group, suggesting the elevation of those markers in HIV+ were likely related to HIV+ status and not to age‐dependent inflammatory status.

### 2.3 Reduction of CD4 T cells and increase of CD8 T cells in PBMCs from HIV+ subjects

**FIGURE 2:**
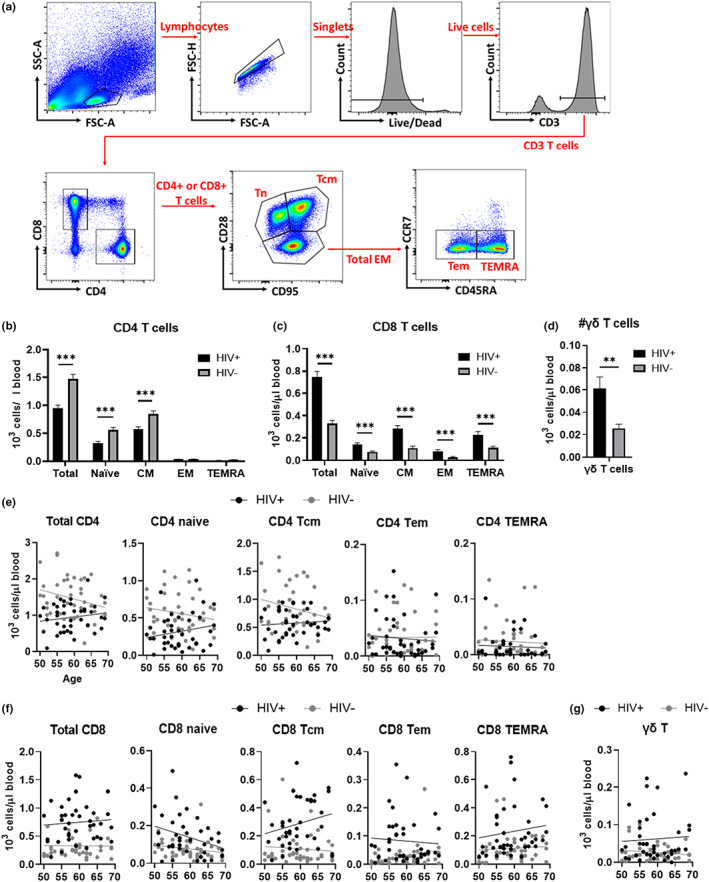
T cell subset analysis in PBMCs from successfully treated HIV‐positive participants (HIV+) and HIV‐negative counterparts (HIV−). (a) Gating strategy for CD4 and CD8 T cell subset analysis by flow cytometry shown in (b). Lymphocyte population without cell debris was selected followed by removal of doublets and dead cells. Single positive CD4 and single positive CD8 cells are selected in CD3+ population and naïve, central memory (CM) and total effector memory (EM) cell populations are gated using CD28 and CD95, respectively. Total EM population was then divided into effector memory (EM) and CD45RA+ EM (TEMRA) using CCR7 and CD45RA. (b–d) Absolute numbers of total, naïve, central memory (CM), effector memory (EM) CD4 (b) and CD8 (c) T cell subsets and δγ T cells (d). Means and standard errors in each age/HIV group are shown. Significant differences between HIV+ (black) and HIV− (gray) were determined by unpaired t‐test. *p < 0.05, **p < 0.01, ***p < 0.001. (e–g) analysis of covariance f absolute number of CD4 (e) and CD8 (f) T cell subset and δγ T cells in blood with age for HIV+ (black dots and lines) and HIV− (gray dots and lines), using an F‐test for the difference in slopes. PBMCs, peripheral blood mononuclear cells.

To evaluate T cell homeostasis in HIV+ participants on ART as a function of aging, we first compared the absolute numbers of total T cells and their subsets in the peripheral blood mononuclear cells (PBMCs) from HIV+ and HIV− (gated as in Figure 2a). Absolute number of CD4 T cells were lower and absolute number of CD8 T cells higher in HIV+ compared with HIV−, which resulted in the decrease of CD4/CD8 ratio (Figure 2b,c). These changes were driven by likely HIV‐mediated original depletion of CD4 Tn and Tcm subsets, and by an expansion of CD8 T cell subsets as a likely consequence of direct or bystander stimulation with HIV as well as by reactivating and/or opportunistic infections. The absolute number of γδ T cells was also significantly higher in HIV+ subjects **(**Figure 2d). The regression analysis with age showed that CD4 T cell populations of HIV+ and HIV− may have different regression patterns with increasing age, specifically total, naive, and Tcm populations (Figure 2e); however, none of these differences were statistically significant. Age‐dependent decrease of CD8 Tn cells, which have been reported before, were observed in both HIV+ and HIV−, but the decrease is more extensive in HIV+ (slope: −0.006131, *p* = 0.0483) compared with HIV− (slope: −0.001983, *p* = 0.1840). Of interest to our hypothesis, similar to the inflammatory marker changes, the changes in absolute numbers of CD4 and CD8 between HIV+ and HIV were less significant in the older age group, probably due to the overall age‐related lymphopenia. The fact that aging did not potentiate the effects of HIV, but rather ameliorated the phenotype observed in HIV+, was surprising, and could suggest the frequency of cell phenotypes are affected differently by HIV infection and by age.

### 2.4 Expression of functional markers on T lymphocytes on successfully treated HIV‐positive subjects

**FIGURE 3:**
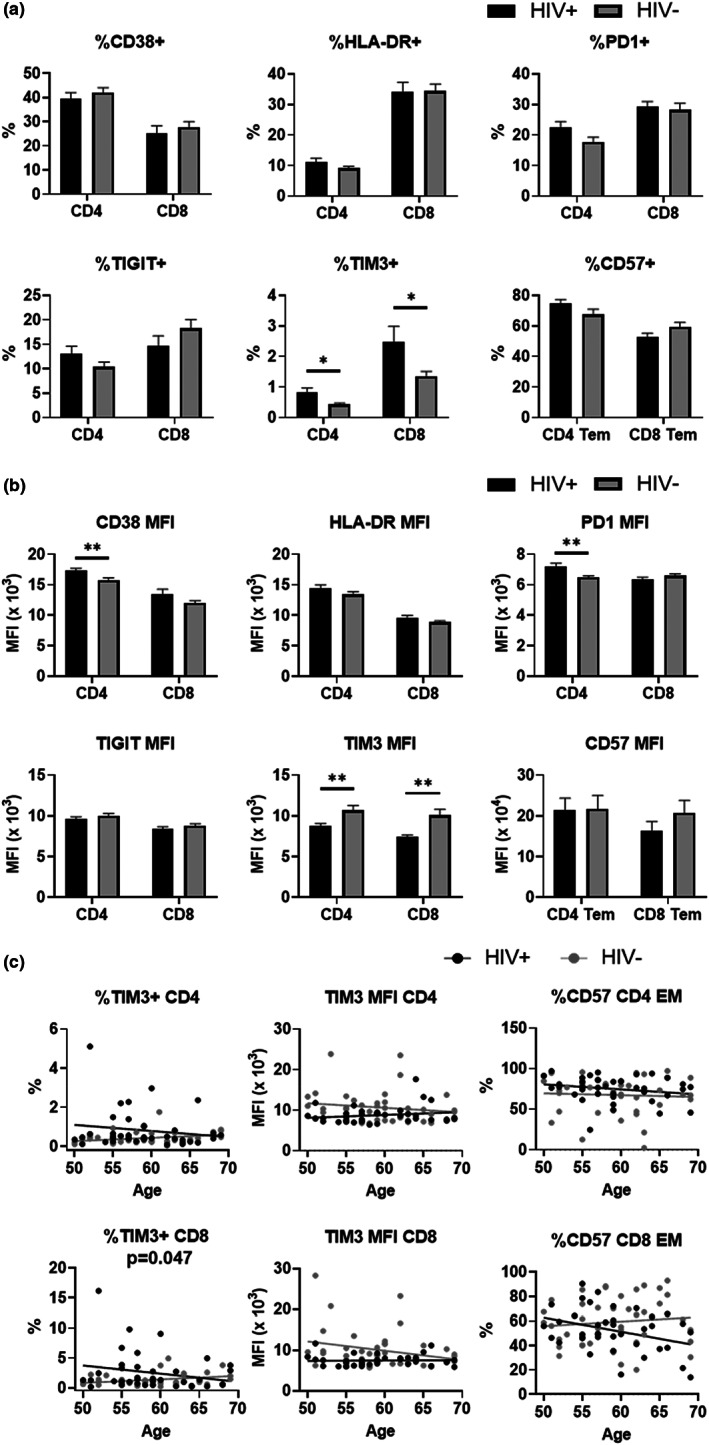
Expression of functional markers on CD4 and CD8 T cells in PBMCs from HIV+ and HIV−. (a) Frequency of each functional marker positive cells in total CD4 or CD8 T cells. (b) Mean fluorescence intensity (MFI) of functional marker signals in each respective positive populations of CD4 or CD8 T cells. Means and standard errors in each HIV group are shown. Significant differences between HIV+ (black) and HIV− (gray) in each age were determined by unpaired t‐test. *p < 0.05, **p < 0.01, ***p < 0.001. (c) Regression analysis of frequencies of TIM3 positive cells and MFI of TIM3 signals in TIM3 positive population of CD4 and CD8 T cell in blood with age for HIV+ (black dots and lines) and HIV− (gray dots and lines). *p* Values are from an F‐test statistic for the difference in slopes. PBMCs, peripheral blood mononuclear cells.

Expression of functional activation and inhibitory/exhaustion markers on CD4 and CD8 T cells was examined to investigate the status of T lymphocytes in successfully treated HIV+. CD38 and HLA‐DR have been recognized as activation markers expressed on activated cells during active microbial infection (Rodrigues et al., 2002). We found no difference in the frequencies of CD38+, HLA‐DR+ cells in CD4 or CD8 T cell subsets between HIV+ and HIV− (Figure 3a). The mean fluorescent intensities (MFIs) of CD38 and HLA‐DR levels were equivalent in HIV+ and HIV− participants, on average. Although the increase in MFI of CD38+ CD4 T cells in HIV+ compared with HIV− was statistically significant, the absolute difference was minimal. We further evaluated expression of PD‐1, TIM3, and TIGIT as inhibitory markers, also expressed on exhausted T cells. We found no differences in the frequency of PD‐1+ or TIGIT+ cells in CD4 or CD8 T cells between HIV+ and HIV− (Figure 3a). MFIs of PD‐1+ or TIGIT were also at the same levels in HIV+ and HIV−, with exception of PD‐1 MFI, which increased in HIV+ compared with HIV− (Figure 3b). Similar to CD38 MFI, the average increase in PD‐1 MFI was minimal and may not have impact on T cells function. By contrast, HIV+ T cells contained a significantly higher frequency of TIM3+ cells in both CD4 and CD8 T cells compared with HIV− counterparts (Figure 3a). In spite of the increase in TIM3+ cell frequency, its MFI in TIM3+ CD4 or CD8 cells decreased in HIV+ participants (Figure 3b). We further analyzed the relationship of TIM3 expression and age (Figure 3c) and found the frequency of TIM3+ in CD4 from HIV+ inversely correlate with age, while the frequency of TIM3+ in CD4 from HIV− positively correlate with age (*p* = 0.0469). A similar pattern was observed in CD8, although this was not statistically significant. MFI of TIM3 estimates demonstrated an opposite pattern from the frequencies, in both CD4 and CD8, TIM3 MFI in HIV+ was positively correlated with age, while negatively correlated in HIV−; these correlations were not statistically significant from zero.

CD57 is expressed on terminally differentiated cells, characterized by high levels of cytotoxicity and cytokine secretion and reduced or absent proliferation in response to TCR stimulation. The increase of CD57+ cells is often considered a correlate of T cell cellular senescence. There was no difference in either the mean frequency or MFI of CD57+ cells in both CD4 and in CD8 between HIV+ and HIV− (Figure 3a,b) in our study participants. However, the frequency of CD57 in CD8 is negative correlated with age in HIV+ (*R* = −0.352, *p* = 0.028), and positively correlated with age in HIV− (*R* = 0.103, *p* = 0.515), although not statistically significant (Figure 3c).

Overall, the pattern of phenotypic changes is increased expression of inhibitory/exhaustion‐related markers in HIV+ participants. Consistent with our other findings, such differences are more prominent in the younger age, and the differences between HIV+ and HIV− diminished (or reversed some cases) with increasing age.

### 2.5 T cell proliferation and functional markers on non‐proliferative and proliferative cells

**FIGURE 4:**
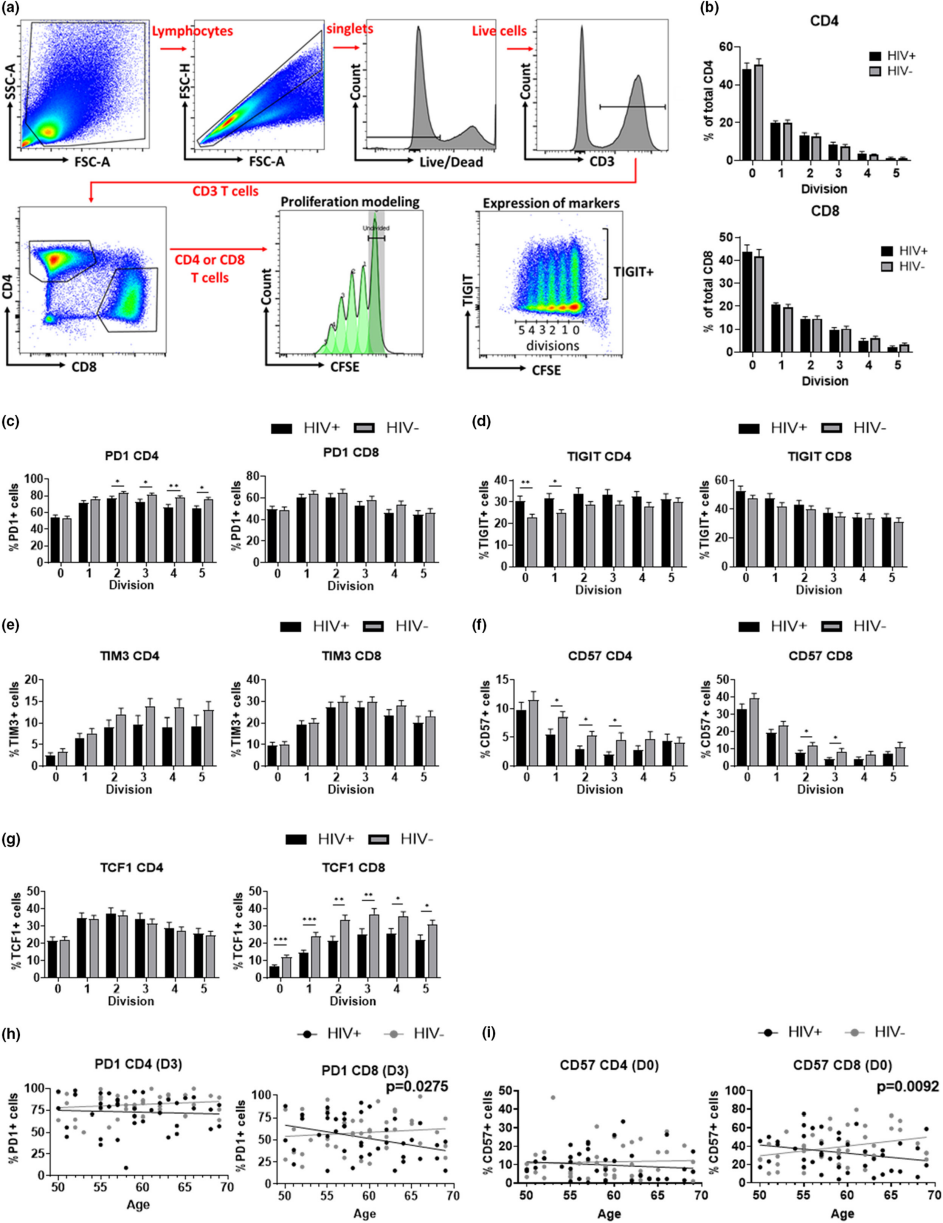
T cell proliferation and marker expression profiles in non‐dividing and dividing CD4. And CD8 T cells after CD28/CD3/CD49d stimulation in HIV+ and HIV−. (a) Gating strategy for non‐dividing and dividing CD4 and CD8 T cells using proliferation modeling with FlowJo. The low‐right panel shows gating example of dividing cells and positive populations on TIGIT. (b) Number of cells in each division. (c–g) frequencies of PD‐1+ (c), TIM3+ (d), TIGIT+ (e), CD57+ (f) and TCF‐1+ (g) cells in each divisions. Means (columns) and standard errors (bars) in each HIV group are shown. Significant differences between HIV+ (black) and HIV− (gray) were determined by unpaired t‐test. *p < 0.05, **p < 0.01. (h,i) analysis of covariance of frequencies of PD‐1+ cells in CD4 and CD8 (h) cells or frequencies of CD57+ cells in CD4 and CD8 T cells with age. *p* Values are the significance from an F‐test statistic for the difference in slopes.

We stimulated PBMC with the CD3/CD28/CD49d antibody cocktail and labeled with CFSE to analyze T cell proliferative ability as well to evaluate functional characteristics of T cell subsets in successfully treated HIV+ population as a function of aging (Figure 4a). Approximately 50% of CD4 T cells and 60% of CD8 T cells underwent at least one cell division. The number of proliferating cells did not differ between HIV+ and HIV− in either CD4 and CD8 T cells, indicating that T cell in successfully treated HIV+ participants retain proliferative capacity at least under our experimental conditions (Figure 4b). Analysis of expression of activation/inhibitory markers on cells at different number of cell divisions revealed that both PD‐1+ cells and TIM3+ cells were less frequent among CD4 and CD8 cells that divided two or more times, specifically in HIV+ participants (Figure 4c,e). Careful examination of the data suggested, however, that cells from HIV+ participants exhibited relatively high levels of PD‐1 in the first division, but that these levels were not sustained in more advanced division, unlike in their HIV− counterparts (Figure 4c). Whether and to what extent that may belie different signaling via the TCR in HIV+ vs. HIV+ participants remains to be examined. Induction of TIM3 was more gradual in both HIV+ and HIV− groups, being again similar in the first division, but then remaining constant on cells from HIV+ participants, and further increasing to much higher levels in the HIV− group (Figure 4e), suggesting different regulation of these two markers and their different dysregulation in HIV+ carriers. The expression of TIGIT did not follow this pattern and exhibited somewhat increased levels in non‐dividing and early‐dividing CD4 T cells of HIV+, but here HIV cells caught up with TIGIT expression in the more advanced division (Figure 4d). This difference was not observed in CD8 T cells. The frequency of PD‐1+ in dividing CD8 T cells was associated with age in different way in HIV+ and HIV− (Figure 4h). On average, the frequency of PD‐1+ CD8 increases with increasing age in HIV− participants but decrease with increasing age in HIV+ participants (*p* = 0.0224).

Not surprisingly, the expression of CD57+ declined in proliferating T cells, and the kinetics of the decline was often steeper in the HIV+ group compared with HIV (Figure 4f). The association between the frequency of CD57+ in non‐dividing CD8 cells and age was statistically significantly different between HIV+ and HIV− participants; increasing in HIV− participants while increasing in HIV+ participants (*p* = 0.0093) as observed in PD‐1 (Figure 4i). TCF‐1 is a self‐renewing marker and its expression positively correlates with the replicative ability of T cells (Kratchmarov et al., 2018). The TCF‐1+ CD8 T cells were less abundant in HIV+ compared with HIV− group, consistent with the idea that the CD8 T cell compartment was stimulated more intensely in the HIV+ participants (Figure 4g). These results demonstrated that even though the bulk proliferative ability upon the stimulation did not appear to be altered by ART‐controlled HIV infection, the proliferating cells exhibited increased expression of late differentiation markers. This suggests that T cells are altered during aging in the successfully treated HIV+ participants in numerous and subtle ways relative to HIV− counterparts.

### 2.6 HIV+ individuals does not have significant DNA damage for shortening of telomere compared to HIV−

To evaluate aging phenotypes in immune cells at the DNA level, we measured gamma H2A.X, which are indication of DNA damage (Fernandez‐Capetillo et al., 2004). Gamma H2A.X‐positive cells are surprisingly higher in HIV− CD4 Tem and CD8 Tem than in HIV+ (Figure S1) and not associated with age in either groups.

We also measured telomere length of T cells by telomere peptide nucleic acid (PNA) probes. Telomeres are repeating hexametric sequences of nucleotides at chromosomal ends that provide the stability and that shorten with each replication. The mean fluorescence index (MFI) of PNA signals in both CD4 and CD8 T cells were not statistically significant (Figure S2).

As conclusion, we did not observe aging phenotypes at the DNA levels in HIV+ individuals compared with HIV− in this study.

### 2.7 HIV+ individuals have higher humoral and cellular immune response against CMV

**FIGURE 5:**
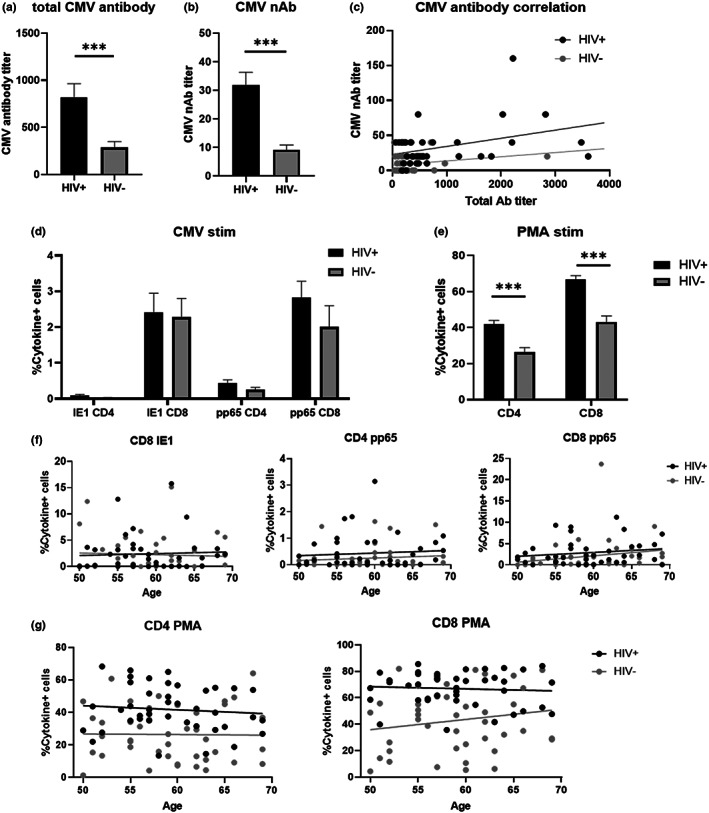
Host response to CMV‐specific and non‐specific stimulation. (a,b) Total antibody titers (a) and 80% neutralization titers (b) against CMV in plasma. Significant differences between HIV+ (black) and HIV− (gray) antibody titers were determined by unpaired t‐test. ***p < 0.001. (c) Correlation between total anti‐CMV antibody (tAb) titer and anti‐CMV neutralization antibody (nAb) titer in HIV+ and HIV−. (d) Frequency of IFN‐γ + and/or TNF‐α + cells after 3 h of CMV‐peptide stimulation of PBMCs from HIV+ and HIV−. (e) Frequency of IFN‐γ + and/or TNF‐α + cells after 3 h of PMA/ionomycin stimulation of PBMCs from HIV+ and HIV−. Statistical significance between frequencies of cytokine‐positive cells in HIV+ and HIV− were determined by unpaired t‐test. ***p < 0.001. (f,g) Analysis of covariance showing statistical significance from an F‐test statistic for the difference in slopes in CMV‐peptide stimulated cells (f) and PMA/ionomycin stimulated cells (g). PBMCs, peripheral blood mononuclear cells.

We took advantage of the fact that all our participants were hCMV‐seropositive to examine how immune responses to CMV may be affected by HIV status and treatment and aging. hCMV‐specific total antibody titers in plasma were statistically significantly higher in HIV+ compared with HIV− (Figure 5a). We devised a hCMV fluorescence‐based neutralization assay and determined the neutralizing antibody (nAb) titers to examine functional capabilities of anti‐hCMV humoral responses, this is described in the materials and methods. hCMV nAb titers remained constant and generally between low and negative in the HIV− participants, even though all participants had overall antibody titers (Figure 5b). HIV+ participants had significantly higher nAb titers than their HIV− counterparts, and the difference appeared to further increase with age (Figure 5b), although the difference is not statistically significant. Furthermore, total antibody titer and nAb titer were not correlated in HIV− (*R* = 0.219, *p* = 0.143) but were significantly correlated in HIV+ (*R* = 0.377, *p* = 0.014) (Figure 5c); we note that the direction of the correlation was positively correlated in both HIV− and HIV+ participants.

Given that the primary control of reactivating hCMV is under purview of T cells, we examined T cell cytokine secretion after both hCMV‐specific (IE1 and pp65 peptide pools) and polyclonal, non‐specific stimulation with phorbol ester and calcium ionophore (PMA and ionomycin). We measured IFN‐γ and TNF‐α expression by intracellular cytokine staining and scored the cells expressing either one or both cytokines as responsive. Cytokine responses of CD4+ cells against IE1 were at the background level (without stimulation). No significant difference was observed in % T cell reactivity to CMV peptides in either CD4 or CD8 T cells from HIV+ and HIV− participants (Figure 5d), and there were no significant associations with CMV‐specific T cell reactivity with increasing age (Figure 5f).

The response of T cells to PMA/ionomycin revealed higher cytokine responses in HIV+ than the HIV− participants, a result that was consistent in both CD4 and CD8 (Figure 5e). This broad increase in cytokine‐secreting T cells may contribute to the proinflammatory status in HIV+ participants but does not appear to be age‐sensitive, at least in our participant cohort (Figure 5g).

### 2.8 HIV+ display distinct correlation pattern between inflammatory signatures and immune phenotypes different from HC

**FIGURE 6:**
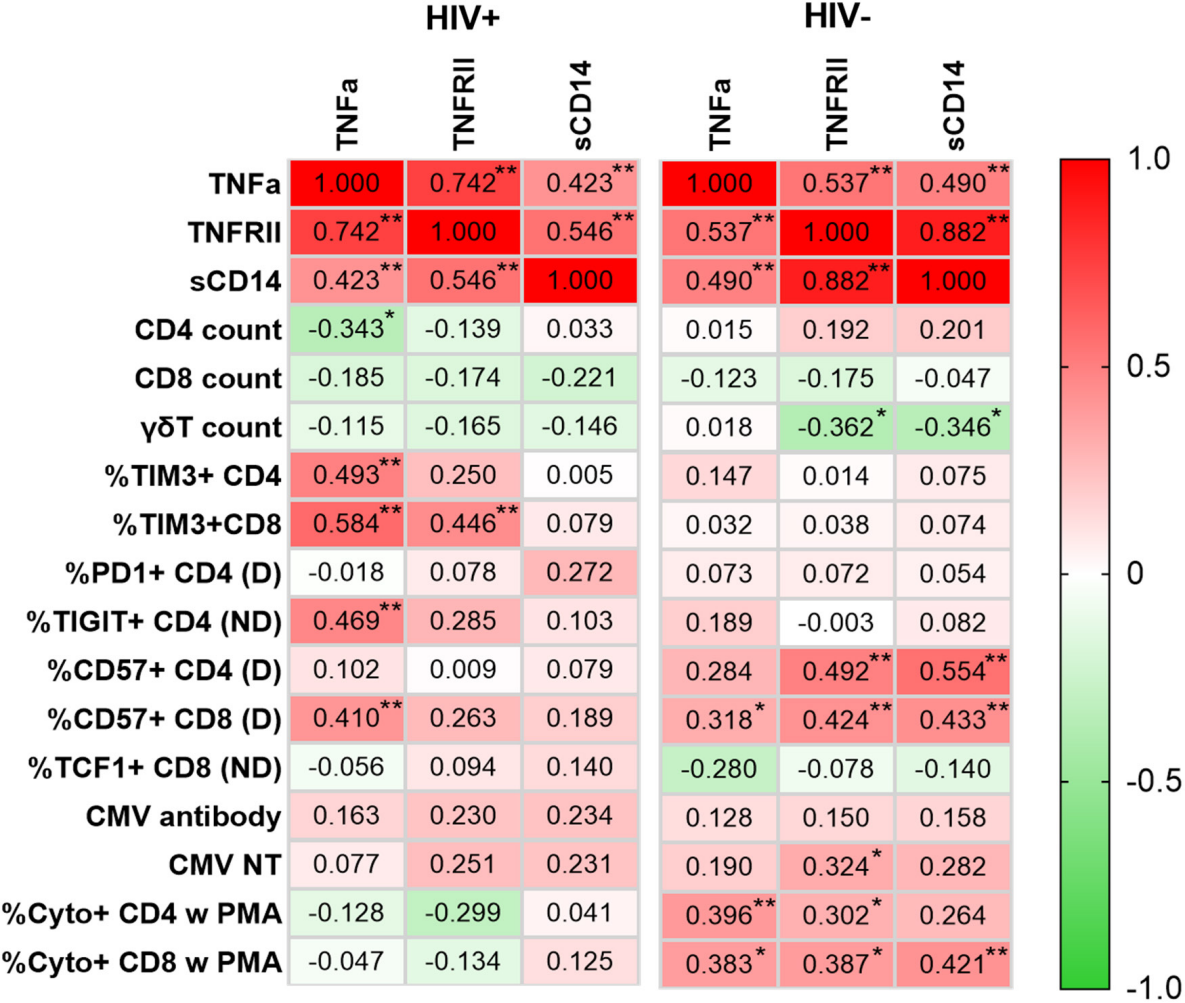
Heatmap of the bivariate Pearson correlation matrix. Correlations between inflammatory markers and other significant parameters in HIV+ and in HIV− are shown. Red and green colors represent positive and negative correlation, respectively, and darker colors representing larger values. Pearson's *r* are shown in the boxes with statistically significant *p*‐values (represented by stars) based on a z‐test statistic for each correlation under the hypothesis of difference from zero. *p < 0.05, **p < 0.01. D, Dividing cells; ND, Non‐dividing cells.

Finally, we analyzed the correlation between inflammatory markers and other variables to explore the landscape of immune status in HIV+ participants on successful ART. Figure 6 shows the correlation between the variables in HIV+ and HIV− participants. The plasma inflammatory markers were correlated regardless of HIV status. Some correlations greatly differed between HIV+ and HIV− participant groups. Inflammatory markers showed strongly negative correlation with cytokine expression from T cells after the stimulation in HIV− but were positively correlation in HIV+. TNF‐α strongly positively correlated with TIM3 expressions on T cells and TIGIT expression on non‐dividing CD4 T cells after the stimulation, and negatively correlated with CD4 count in HIV+. Other pattens include that the correlation of TNFRII and sCD14 with other variables were of different correlative direction from TNF‐α, and strong correlation with CD57 expressions in dividing cells after stimulation was observed.

## 3 DISCUSSION

HIV infection causes a massive and catastrophic disruption in the host immune system and mucosal homeostasis, which in subjects on ART resemble some of the immune changes found in physiological aging. It remains difficult to prove whether those homeostatic disturbances in HIV+ participants actually represent accelerated aging or whether they only result in phenotypes similar to aging. Active HIV infection causes many changes in immune phenotypes such as CD4 T cell death, T cell activation and exhaustion and expression of inflammatory markers. Those changes are significantly reduced with ART, yet chronic diseases abound in this group of patients and appear earlier than in their HIV‐negative counterparts. Some of diseases were attributed to ART toxicity especially in the early days of ART (Group et al., 2007; Ryom et al., 2013; Scherzer et al., 2012), but the ART has been improved and the increase of those diseases over time does not exclude low HIV replication as a cause. The chronic diseases in HIV‐positive patients with successful ART correlates with inflammatory markers, while those markers do not correlate with duration of ART (Burdo et al., 2011; Kuller et al., 2008; Sandler et al., 2011). Therefore, the high mortality and morbidity by non‐AIDS related disease in HIV‐positive patients with ART is more likely due to HIV infection itself, rather than ART toxicity.

In this study, we performed immune profiling of successfully treated (at least 5 years of ART and <50 copies HIV RNA per ml plasma) HIV+ participants and age‐matched HIV‐negative controls, all seropositive for hCMV. Our focus in this study was to understand how virologically undetectable HIV infection affects immune aging under successful ART. We were somewhat surprised to find that systemic inflammatory markers in those individuals were subtly and not very predictably elevated, and in particular that IL‐6 and CRP, the two stalwart markers of elevated inflammation with aging, did not change in an age‐related manner over the span of two decades in our cohort. However, our data were similar to the findings of de Armas et al., who compared plasma inflammatory cytokines from successfully treated HIV+ with those from HIV− in different age groups and found only TNFR2 was elevated in HIV+ in all age groups while IL‐6 and IL‐8 were actually decreased in HIV+ compared with HIV− (de Armas et al., 2017). Moreover, Margolick et al. reported that TNF‐α was the only analyte out of 17 measured cytokines or chemokines (including IL‐6, IL‐8, and CRP) which showed significant increase in successfully treated HIV+ compared with HIV− participants (Margolick et al., 2018). In our study, the only sustained inflammation‐related marker elevated across all ages (50–70) in HIV+ participants was sCD14, generally viewed to be a surrogate marker for microbial translocation. The persistent elevation of sCD14 after years of ART treatment (Mendez‐Lagares et al., 2013) and association of sCD14 plasma level and all‐cause mortality in HIV+ with ART (Sandler et al., 2011) suggests the sCD14 may be a biomarker of chronic diseases in HIV+ with ART, and, perhaps, that impaired gut barrier function may be one of the factors driving these conditions.

Major T cell subsets were predictably reduced (CD4 Tn and Tcm) or elevated (all CD8 subsets) with successfully treated HIV infection, and these changes showed no age‐sensitivity. T cell phenotypes in successfully treated HIV+ participants were not very different from those in HIV−, lacking signs of massive activation or massive cellular exhaustion, and only showing increase in number of inhibitory marker positive cells. The increase in those markers at homeostatic T cells diminished in older population in both groups. In contrast, response to polyclonal stimulation were generally decreased in HIV+ compared with HIV+, exception of TIGIT, which is increased in HIV+. Again, contrary to homeostatic T cells, the magnitude of the difference between HIV+ and HIV− in stimulated T cells were greater with increasing age.

Immune response to hCMV was more informative and insightful and suggested impaired control of hCMV in HIV+ participants, with possible increased rate of virus reactivation based on the fact that HIV+, but not HIV−, participants exhibited significant increase in anti‐hCMV neutralizing antibodies, and that this increase was more pronounced with increasing age. T cell responses to hCMV immediate‐early antigen 1 (IE1) regardless of the HIV status or age. As its name states, IE1 is among the earlies transcripts of hCMV and is transcribed early both in the course of primary infection and reactivation (Ye et al., 2020), explaining ubiquitous and strong T cell stimulation by this antigen. T cell responses against pp65, a late‐transcribing major tegument phosphoprotein (Ye et al., 2020), were more pronounced in younger HIV+ participants, consistent with the idea that the higher cytokine response in HIV+ participants likely reflects strong hCMV reactivation/ precipitated by the HIV infection itself and not mediated by ART therapy. The finding that older HIV− participants “catch up” with HIV+ participants in their T cell anti‐pp65 responses is likely reflective of the repeated rounds of hCMV subclinical reactivation over time in both groups. Finally, analysis of cytokine production following polyclonal stimulation revealed strong, persistent propensity of T cells from HIV+ participants to produce significantly more inflammatory cytokines (IFN‐γ and TNF‐α) regardless of age.

Correlational analysis stratified by HIV status allowed us to evaluate distinct correlation profiles between inflammatory markers, T cell counts and frequencies, phenotypes, and functional responses in successfully treated HIV+ and HIV− participants. The inflammatory markers elevated in HIV+ did not correlate with T cells responses, but positively correlated with frequency of inhibitory factor/exhaustion markers (TIGIT and TIM3) on CD8 cells, and negatively correlated with CD4 counts, consistent with damage to CD4 cells, exhaustion of CD8 cells, and incompletely repaired gut barrier. This pattern of correlation was dramatically different in HIV− participants, where inflammatory cytokines positively correlated with T cell responses to polyclonal stimulation and to expression of CD57, suggesting qualitative difference in immune system regulation.

Limitations of this study are primarily twofold. First, the study is of relatively low power, with 42–46 subjects/group, which impacted finding statistical significance even when differences were visually apparently. Second, the age‐related conclusions and the results on age‐sensitive traits are limited by a relatively narrow span of ages, from 50–69 years of age. The fact that over this range of ages we did not observe changes in certain well‐known age‐sensitive parameters, like IL‐6 and CRP levels, may highlight that type of limitation. We also would like to note that the difference we see in this paper may not directly be caused by HIV infection but caused by secondary changes due to the HIV infection, such as more frequent reactivation of CMV in HIV+ as the higher anti‐CMV antibody titers in HIV+ suggest. However, even with these limitations, we have identified two sustained and mechanistically potentially different contributors to increased systemic inflammation in HIV+ participants, with a potential to exacerbate multiple chronic diseases. These include (i) the persistent significantly higher elevation of sCD14; and (ii) increased frequencies of T cells producing inflammatory cytokines (IFN‐γ and TNF‐α) upon polyclonal stimulation. Both were elevated across both ages examined, suggesting prolonged disturbances in gut mucosal integrity and potential microbial translocation, and a linked, or independent/additive, chronic “trigger‐happy” inflammatory T cell accumulation, both of which could contribute to the adverse effects of poorly controlled inflammation and its impact on various chronic diseases (Bektas et al., 2018; Sandler & Douek, 2012). Finally, survivor bias is a possibility in the older successfully treated HIV+ population, particularly, those who have been on active treatment long after initial diagnosis, as many in the 60+ age group did not have access to treatment when/if exposed when they were younger.

## 4 EXPERIMENTAL PROCEDURES

### 4.1 Human subjects and sample preparation

This study was approved by the Institutional Review Boards (IRB) at the University of Arizona (IRB protocol#2102460536). HIV‐positive subjects, who are treated successfully with ART (HIV RNA <50 copies/ml in plasma), were recruited from Banner‐University Medicine North HIV‐positive study cohort was selected from HIV‐positive males, who diagnosed more than 5 years ago, and are over 50 years old of age. Age‐ and sex‐matched controls are healthy community‐dwelling individuals, recruited in Arizona. CMV seronegative individuals were removed from the cohort since CMV infection affects many aspects of immune phenotype. Demographic information of the cohort is shown in Table 1. Blood for complete blood count was collected in BD vacutainer with EDTA and submitted to Sonora Quest. Blood for peripheral blood mononuclear cells (PBMCs) and plasma was collected in BD Vacutainer with sodium heparin. Plasma was separated by centrifugation at 1100 *g* for 10 min and PBMC was isolated from the buffy coat by Ficoll‐Paque PLUS (GE Healthcare) and cryopreserved in fetal bovine serum (FBS) + 10% DMSO.

### 4.2 Measurement of inflammatory markers

Interleukin‐6 (IL‐6), Interleukin‐8 (IL‐8) and tumor necrosis factor (TNF)‐α (HCYTOMAG‐60 K and HSTCMAG‐28SK), TNF receptor (TNFR) 1 and 2 (HSCRMAG‐32 K), soluble CD14 (sCD14) (HCVD6MAG‐67 K), and C‐reactive protein (CRP) (HNDG2MAG‐36 K) in plasma were quantified by Millipore MagPix kit (Millipore) and soluble CD163 in plasma was quantified by ELISA kit (Invitrogen EHCD163) according to the manufacturer's protocol.

### 4.3 Antibody titers against CMV

Serum samples with high IFA‐scored antibody titers (i.e., 2560), obtained from prior studies, were used as the top standards for CMV as previously described (Stowe et al.,  2014). Twofold serial dilutions of the standards (2560, 1280, 640, 320, 160, 80, 40, and 20) were made with PBS in separate tubes. One hundred microliters of positive and negative controls, standards, and diluted patient samples (all dilutions were at 1:101 with PBS) were pipetted in duplicate into individual microplate wells followed by a 30 min incubation (all steps were carried out at room temperature). The plates were then washed 3 times with 350 μl wash buffer using an Embla microplate washer (Molecular Devices). Next, 100 μl of enzyme conjugate (peroxidase labeled anti‐human IgG) was pipetted into the wells followed by another 30 min incubation period. The plates were then washed 3 times, and 100 μl of chromogen substrate (TMB/H2O2) was pipetted into the wells. The plates were then covered to protect from direct light and incubated for 15 min. One hundred microliters of 0.5 M sulfuric acid was added to each well to stop the reaction. Absorbance was then read at 450 nm (reference wavelength 620 nm) using a SpectraMax Plus 384 (Molecular Devices). The values of the unknown samples were assigned in relation to the standard curve.

### 4.4 Neutralization antibody titer to CMV

Human foreskin fibroblast (HFF)‐1 was purchased from ATCC (#SCRC‐1041). HFF‐1 cells were grown in complete DMEM (4.5 g glucose/L, 4 mM L‐glutamine, 1 mM Sodium Pyruvate, 1X Penicillin/Streptmycin and 15% FBS). GFP‐expressing hCMV virus was kindly provided by Dr. Felicia Goodrum (University of Arizona) (Umashankar et al., 2011). Human plasma from each subject was diluted in complete medium at 1:10, followed by twofold dilutions 5 times. The diluted plasma was then mixed with same volume of medium containing 800 infectious unit (IU) of virus and incubated for 2 h at room temperature. HFF‐1 monolayer in a 96‐well plate was infected with half amount of the plasma/virus mixture. No plasma control wells were infected with medium containing 400 IU of the virus. The plate was incubated in 37°C CO_2_ incubator for 48 h. The number of GFP‐positive cells per field was counted by Cytation 5 (Biotek). Cutoff value was calculated as 20% of no plasma control, which represent 80% reduction of the infection. The highest dilution rate, which exceeded 20% positive, was read as a neutralizing titer.

### 4.5 PBMC stimulation and flow cytometry

Cryopreserved PBMCs were thawed in RPMI medium supplemented with 10% FBS, penicillin, and streptomycin in the presence of DNAse (Sigma), rested overnight in X‐Vivo medium (Lonza) supplemented with 5% human male AB serum. Antibodies used for flow cytometry is listed in Table S1. For proliferation experiment, PBMCs were stained with CFSE Cell Proliferation kit (Invitrogen) and cultured in a plate coated with antibodies against CD3 (BioLegend), CD28 (eBioscience) and CD49d (BD Pharmingen), in the presence of IL‐2 (TECIN™ Teceleukin, National Cancer Institute) at 100 unit/ml for 5 days. Fresh medium was added every 2 days. Following incubation, cells were stained with surface staining antibodies for 1 h in PBS (Lonza) + 2% FCS at 4°C. Dead cells were stained with Live/Dead Fixable Blue Dead Cell Stain Kit (Invitrogen) and cells were fixed and permeabilized with FOXP3/Transcription Factor Staining Buffer Set (Invitrogen) for intracellular staining (ICS) of TCF‐1. For CMV peptide or PMA/Ionomycin stimulation, PBMCs were stimulated with CMV pp65 and IE1 PepTivators (Miltenyi Biotec) or Cell Stimulation Cocktail (Invitrogen) in the presence of Brefeldin A (Millipore Sigma) for 4 h. Dead cells were stained with Zombie Aqua (BioLegend) and all cells were fixed and permeabilized with BD Cytofix/Cytoperm (BD) for ICS of cytokines.

The subsets of T cells are defined as followed; Naive (Tn): CD28^int^CD95^low^; central memory (Tcm): CD28^high^CD95^high^; effector memory (Tem): CD28^low^CD95^high^CCR7^−^CD45RA^−^ terminally differentiated EM (Te); CD28^low^CD95^high^CCR7^−^CD45RA^+^ (Figure 1a). The gate for expression of functional markers were set by using a fluorescence minus one controls. Samples were acquired using a Cytek Aurora cytometer (Cytek) and analyzed by FlowJo™ v10.7.2 Software (BD Life Sciences).

### 4.6 Multicolor fluorescence in situ hybridization for determination of telomere length

Experiment was performed as described before (Pulko et al., 2016). Briefly, PBMCs were first stained with LIVE/DEAD Fixable Dead Cell Stain and then incubated with antibodies to stain the surface markers (Table S1). Cells were fixed and permeabilized with BD Cytofix/Cytoperm (BD). Samples were than washed in PBS, fixed in 1 mM BS3 (ThermoFisher Scientific) and quenched with 50 mM Tris–HCl in PBS (pH 7.4). The cells were washed in PBS, and then in hybridization buffer (70% deionized formamide, 28.5 mM Tris–HCl pH 7.2, 1.4% BSA and 0.2 M NaCl). The samples were subsequently resuspended in hybridization buffer and incubated with of the PNA TelC –Alexa Fluor 647 probe (PNA Bio Inc.) and heated for 10 min at 82°C, rapidly cooled on ice and left to hybridize for 1 h at room temperature in the dark. Lastly, the samples were washed in post‐hybridization buffer (70% deionized formamide, 14.25 mM Tris–HCl pH 7.2, 0.14% BSA, 0.2 M NaCl and 0.14% Tween‐20) and in PBS 2% BSA before acquisition on Cytek Aurora cytometer (Cytek) and analyzed by FlowJo™ v10.7.2 Software (BD Life Sciences).

### 4.7 Statistics

Unpaired t‐test was used to compare means and Chi‐Square test was used to analyze categorical values in the demographic table. Analysis of covariance was used to evaluate HIV status moderation of the relationships between immunologic measures and age. The F‐test statistic was used in a stepwise fashion to test interactions, then common slopes with different intercepts and a common slope models. The z‐test statistic was used to test Pearson correlations (*ρ*) under a null hypothesis of zero correlation. Software included R version 4.4.2 and IBM SPSS version 28.

## AUTHOR CONTRIBUTIONS

M.W., M.J., L.D., M.J.S., and R.P.S. conducted experiments and acquired data. M.J., M.J.S., B.J.L., and J.N.Z. contributed to study design. M.W. and B.J.L analyzed and interpreted the data. M.W. Y.C. and C.M. performed recruitment, specimen collection and database maintenance. M.W., E.K.H., B.J.L, and J.N.Z. contributed to writing the manuscript.

## FUNDING INFORMATION

Supported by the USPS Awards AG054317 and AG020719 and the Bowman Professorship in Medical Sciences to J.N‐Ž.

## CONFLICT OF INTEREST

The authors declare that there is no conflict of interest.

## PATIENT CONSENT

All patients provided written informed consent to the study.

## Supporting information


Appendix S1

## Data Availability

The datasets generated during and/or analyzed during the current study are available from the corresponding author on reasonable request.
