# Supplementary material for: Inflammatory and immune markers in HIV‐infected older adults on long‐term antiretroviral therapy: Persistent elevation of sCD14 and of proinflammatory effector memory T cells
Source: Aging Cell. 2022 Aug 16;21(9):e13681. doi: 10.1111/acel.13681 (PMC9470897; doi:10.1111/acel.13681)
Supplement: Supplementary file 1 — Appendix S1 [file ACEL-21-e13681-s001.docx]

Supplimental Table 1: Antibody List for Flow Cytometry

| Target | Fluorochrome | Manufacturer | Clone | Figures |
| --- | --- | --- | --- | --- |
| CD3 | Brilliant Violet 510 | BioLegend | OKT3 | Fig. 2, S1, S2 |
| CD4 | APC-eFluor 780 | Invitrogen | OKT4 | Fig. 2, 3, S1, S2 |
| CD8b | Brilliant Violet 570 | BioLegend | RPA-T8 | Fig. 2, S1, S2 |
| CD28 | Brilliant Violet 421 | BioLegend | CD28.2 | Fig. 2, S1 |
| CD95 | Brilliant Violet 711 | BioLegend | DX2 | Fig. 2, S1 |
| CD45RA | Alexa Fluor 488 | BioLegend | HI100 | Fig. 2, S1 |
| CCR7 | PE-Cy7 | BioLegend | G043H7 | Fig. 2, 4, S1 |
| HLA-DR | Alexa Fluor 700 | BioLegend | L243 | Fig. 2, S1 |
| CD38 | Brilliant Violet 650 | BioLegend | HB-7 | Fig. 2, S1 |
| PD-1 | APC | BioLegend | EH12.2H7 | Fig. 2 |
| TIGIT | Brilliant Violet 605 | BioLegend | A15153G | Fig. 2, 3, S1 |
| TCR γ/δ | PE-Dazzle 594 | BioLegend | B1 | Fig. 2 |
| TIM-3 | PerCP-Cy5.5 | BioLegend | F38-2E2 | Fig. 2, 3, S1 |
| CD57 | PE | BioLegend | HNK-1 | Fig. 2, 3 |
| CD3 | Brilliant Violet 570 | BioLegend | UCHT1 | Fig. 3 |
| CD8b | Brilliant Violet 785 | BioLegend | SK1 | Fig. 3 |
| TCF1 | Alexa Fluor 647 | BioLegend | 7F11A10 | Fig. 3 |
| PD-1 | Brilliant Violet 421 | BioLegend | EH12.2H7 | Fig. 3 |
| CD3 | Brilliant Violet 570 | BioLegend | UCHT1 | Fig. 4 |
| CD4 | Brilliant Violet 650 | BioLegend | OKT4 | Fig. 4 |
| CD8b | ECD | Beckman Coulter | 2ST8.5H7 | Fig. 4 |
| CD28 | PE | BioLegend | CD28.2 | Fig. 4 |
| CD45RA | APC | BioLegend | HI100 | Fig. 4 |
| CD95 | Alexa Fluor 488 | BioLegend | DX2 | Fig. 4 |
| IFN-γ | Alexa Fluor 700 | BioLegend | 4S.B3 | Fig. 4 |
| TNF-α | Brillian Violet 421 | BioLegend | MAb11 | Fig. 4 |
| PD-1 | APCFire 810 | BioLegend | A17188B | Fig. S1 |
| CD57 | Brillian Violet 785 | BioLegend | QA17A04 | Fig. S1, S2 |
| γH2AX | PE | BioLegend | 2F3 | Fig. S1 |

Fig. S1

**Figure S1.** **DNA damages in T cells subsets of HIV+ and HIV-.** Frequency of γ-H2A.X positive cells in total and subsets of CD4 (left) and CD8 (right) T cells from HIV+ and HIV-. Means and standard errors in each HIV group are shown. Significant differences between HIV+ (black) and HC (grey) in each age were determined by unpaired t-test. *p<0.05.

Fig. S2

**Figure S2. Telomere length of T cells subsets from HIV+ and HIV-.** Telomere length was presented as staining of telomere probes with indodicarbocyanine and peptide nucleic acid (PNA), via fluorescence label intensity based on the primer Tel-C. Mean fluorescence intensity (MFI) of the signal in each T cell populations from each HIV group are shown. Error bars: standard errors. No Significant differences between HIV+ (black) and HIV- (grey) were found by unpaired t-test.
